# Research on Multicamera Photography Image Art in BERT Motion Based on Deep Learning Mode

**DOI:** 10.1155/2022/2819269

**Published:** 2022-04-27

**Authors:** Zhao Zhao, Mingyang Song, Hongyue Tang

**Affiliations:** ^1^School of Fine Arts, Hunan Normal University, Changsha 410006, China; ^2^School of Physical Education, Hunan Normal University, Changsha 410006, China

## Abstract

In order to improve the artistic expression effect of photographic images, this article combines the deep learning model to conduct multicamera photographic image art research in BERT motion. Moreover, this article analyzes the external parameter errors caused in the calibration process and uses the checkerboard in the common field of view to calibrate the spatial coordinates of the corners of the board in multiple camera coordinate systems. In addition, this article aims to match the spatial coordinates of the corresponding points to each other and solve the rotation and translation matrix in the transformation process. Finally, this article uses the LM algorithm to optimize the calibration parameters of the camera and combines the deep learning algorithm to perform image processing. The experimental research results show that the research method of multicamera photography image art in BERT motion based on the deep learning mode proposed in this article can effectively improve the expression effect of image art.

## 1. Introduction

In people's daily life, as one of the main tools for image dissemination, photography has become ubiquitous to record and discover different visual possibilities. However, photography and photographic art are two completely different concepts, and photography is not related to all art. Photography is recognized as a relatively recent thing in the field of culture and art. In the 1970s, a large number of art festivals, periodicals, and galleries were launched in Western countries. Immediately afterwards, some colleges and universities set up professional photography colleges and photography departments. In addition, the research on the history of photography in the academic field is also deepening, a large number of photographic works have been included in the art collection, and more and more artists have begun to create photography. In short, the practice and dissemination of photography are no longer confined to a narrow field of practice but lead to the palace of art and culture. At the same time, people's concept of photography is constantly updated. When photography was invented, it was used only as a service tool. Today, however, it is increasingly being discussed and appreciated as the artwork itself. As a result, the public's attitude towards the practicality of photography has changed, attention has been drawn from the perceptual and rational nature of images, and the production, dissemination, and circulation of photography, as well as the form, value, and use of photography, have also changed accordingly.

Some people simply regard the art of photography as craftsmanship based on the principles of optics, chemistry, and mechanics with purity. To put it more frankly, they believe that photography is a kind of performance art of taking pictures, which leads them to ignore the lofty status of photography and its outstanding contribution to the development history of human society. In addition, the importance of photographic practice is generally ignored by some people, who only pay attention to theoretical knowledge and believe that it is enough to master the classic theories, such as visual aesthetics, cultural studies, and image expression in photographic art, and do not pay attention to photographic practice. These understandings of photography art are one-sided and not objective.

Photography art is a comprehensive artistic behavior. It is inclusive and has different connotations in different situations. Sometimes it pays more attention to the connotation of the technical level, and sometimes it focuses more on the expression of culture and emotion. Today's photographic art is specifically a type of modern plastic art. A camera is a tool for photographic creation. Taking the photographer's creative concept as the basic starting point, they use the camera to take pictures of people or things in the real world and uses certain modern processing methods, perform artistic processing on the photographed things, and finally complete the creation of photographic works of art. Through the works showing the living conditions of human beings in the current society, at the same time, it can express the author's thoughts and feelings. Aesthetic features are more of an attribute, the presentation of their own aesthetic features. Photography, as one of many art categories, has aesthetic characteristics similar to other aesthetic activities, that is, the commonality of photography aesthetics and other aesthetic activities. In addition, photography art also has its own characteristics; it belongs to a branch of visual plastic art.

This article combines the deep learning model to study the multicamera photographic image art in BERT motion to improve the performance of photographic image art.

## 2. Related Work

From the perspective of the development process of image text description, it can be divided into three stages: template-based image text description method; retrieval-based image text description method; deep learning-based image text description method [1]. Before the deep learning method was proposed, most of the image description methods used template-based and retrieval-based methods. The template-based image text description method is mainly to annotate the image content, which is based on image annotation technology [2]. Template-based methods rely on visual perception of the relationship between image objects and components and describe images using representations of subject, predicate, environment, and preposition collocations [3]. Reference [4] used the method of image context subject, object, and their relationship to describe the image, used the neighbor similarity algorithm to calculate the matching degree between adjacent tuples, finally calculating the score, and the score is proportional to the matching degree. Reference [5] proposed a Conditional RaIldom Field (CRF) algorithm, the central idea of which is to generate text descriptions for predicted text labels according to template matching rules. Reference [6] improved the template in the image text description task and used the hidden Markov model to fill the template with sentences. Reference [7] applied syntactic analysis to the image text description task, used the VDR (Visual Dependency Representation) method to represent the object relationships contained in the graph with a dependency graph, and then represented the image as a VDR and then traversed the VDR, fully considering the VDR. Syntax tree relationships to fill in gaps in sentences. Template-based image text description methods may be grammatically correct in language descriptions, but the output descriptions are highly template-dependent, have poor generalization performance, and generate text sentences that lack diversity. Due to the backwardness of this method, the image text description task is no longer used [8]. Searching for the relationship between matching text and images is the main purpose of the retrieval-based image description generation task. Retrieval-based image description also includes vision-based retrieval and multimodal-based retrieval methods [9]. The retrieval method based on visual space is to obtain textual information from the features of similar parts in the image. The image retrieval dataset established by the literature [10], for each image, uses appropriate words to describe the image. Reference [11] proposed a large dataset, which includes attribute annotations of objects, which can be used to train attribute classifiers, predicted object attributes, and improved the quality of image text descriptions. The retrieval method based on multimodal space is to perform a multimodal representation of all images and text sentences in the training corpus. For the image to be tested, retrieval is performed in the multimodal space after the image and the text are jointly mapped. First, a set of similar images of the image to be tested is obtained, and the text description corresponding to the test image is obtained according to the text description of the similar image. In [12], the authors proposed to learn multimodal space representation, use kernel function calculation method to extract high-dimensional image features, in the multimodal space represented by image and text jointly, use a sorting algorithm according to the high-dimensional features of images, and find candidate text for the set of similar images of the target. Finally, the candidate text is screened according to a certain sorting algorithm, and the text description corresponding to the image is obtained. Reference [13] applied the neural network with stronger expressive ability in the field of image text description. It makes the image text description generated in the multimodal space more accurate and of higher quality. The retrieval-based method makes full use of the dataset, but the generated image text description largely depends on the dataset. When the gap between the target image and the training data set is large, the effect of the generated text description will be poor, and only the generated text description can be generated. Human-annotated sentences are already in the dataset. Retrieval-based image description methods have better expressiveness, mobility, and practicality. Although some excellent results have been obtained, the retrieval-based method still has dependencies. The production text description is highly dependent on the training corpus, and there are problems such as high complexity, which are seriously affected by human intervention, which makes the generated text description sentences simple and ineffective. With rich semantic information, researchers continue to explore new text description methods [14]. With the popularization of deep learning knowledge, researchers have proposed new methods based on deep learning methods. The advanced and commonly used method is the end-to-end model. On the one hand, deep convolutional neural networks can be used to create models for object features in images; on the other hand, recurrent neural networks can be used to create language models for text [15]. Reference [16] proposed a deep semantic alignment model. The model echoes images and text descriptions by aligning them. In terms of images, a region-based convolutional neural network is used for pretraining, and the features of the images are mapped to the feature space of word vectors to match the two-part features. Descriptions are generated using recurrent convolutional networks in terms of language models. The NIC model based on the English dataset proposed in [17] uses the advanced Inception V3 network to extract image features and uses LSTM to generate descriptions in the language generation model stage. This method plays an important role in the image description task. Reference [18] proposed a hard attention mechanism and a soft attention mechanism, used the combination of the attention mechanism and the LSTM network to obtain the image information of each step, and improved the expressive ability of the image description.

## 3. Global Calibration Optimization of Multicamera System Based on Levenberg–Marquardt Algorithm

Camera calibration has two important functions. One is to construct the relationship between the image plane coordinates and the camera space coordinates, which is explained in detail in the principle of calibration and the principle of binocular stereo imaging. The other is to construct the rotation and translation relationship between the camera coordinate system and other camera coordinate systems.

**Figure 1:**
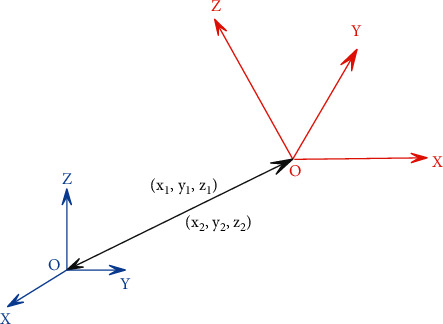
Coordinate system translation.

As shown in Figure 1, there are two coordinate systems in space, (o-x-y-z) and (O-X-Y-Z), and *o* and О are the origins of the two coordinate systems, respectively. Moreover, there is a straight line between the two origins, and the same straight line forms different vectors at different coordinates. Among them, the *o* point and the О point are both (0, 0.0) in their respective coordinates, and the vector from the *o* point to the О point is (*x*_1_, *y*_1_, *z*_1_) . It can also be interpreted as the coordinate of the point О in the (o-x-y-z) coordinate system is (*x*_1_, *y*_1_, *z*_1_).

In the same way, the vector from point О to point *o* is (*x*_2_, *y*_2_, *z*_2_), which is interpreted as the coordinate of point *o* in the (O-X-Y-Z) coordinate system is (x2, y2, 2). Since the directions of each axis corresponding to the two coordinate systems are different, these two vectors are not two opposite vectors, but the length of the same straight line is unchanged. Therefore, the magnitudes of these two vectors are the same, as shown in the following formula:

(1) $$\begin{array}{c} x_{1}^{2}+y_{1}^{2}+z_{1}^{2}=x_{2}^{2}+y_{2}^{2}+z_{2}^{2}. \end{array}$$

A coordinate system with two origins that do not coincide cannot discuss rotation. Therefore, we first move the (o-x-y-z) coordinate system to the vector (*x*_1_, *y*_1_, *z*_1_) until its origin coincides with the origin of the (O-X-Y-Z) coordinate system.

**Figure 2:**
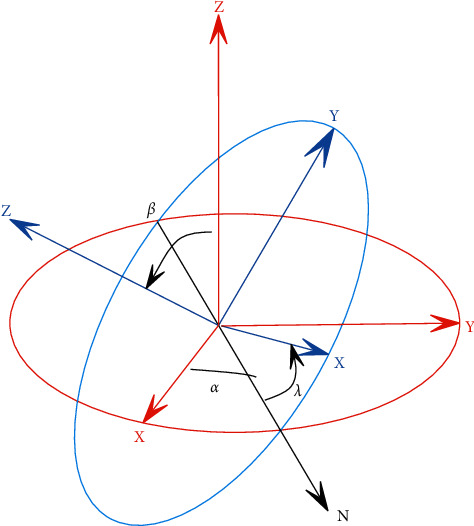
Coordinate system rotation.

As shown in Figure 2, in order to make the two coordinate systems completely coincide, the o-x-y-z coordinates will be rotated around the origin, and the counterclockwise direction is the direction of the rotation angle increment. The process is mainly divided into the following three steps.

The first step, which keeps the *z*-axis stationary, rotates the *x*-axis and the *y*-axis counterclockwise around the *z*-axis by an angle of *a*. At this time, *x* reaches the N-axis position, and a rotation variable *R*_*z*_(*α*) is generated, and its matrix is shown in the following formula:

(2) $$\begin{array}{c} R_{z}\left(\alpha \right)=\left[\begin{array}{ccc} \cos\;\alpha & -\sin\;\alpha & 0\\ \sin\;\alpha & \cos\;\alpha & 0\\ 0 & 0 & 1 \end{array}\right]. \end{array}$$

In the second step, it keeps the *x*-axis stationary, which is the current N-axis, and the *y*-axis and the *z*-axis rotate counterclockwise around the *x*-axis by an angle of *ß*. The *z*-axis just coincides with the *z*-axis, and a rotation variable *R*_*x*_(*α*) is generated, whose matrix is shown in the following formula:

(3) $$\begin{array}{c} R_{x}\left(\alpha \right)=\left[\begin{array}{ccc} 1 & 0 & 0\\ 0 & \cos\;\beta & -\sin\;\beta \\ 0 & \sin\;\beta & \cos\;\beta \end{array}\right]. \end{array}$$

The third step, which again keeps the *z*-axis stationary, rotates the *x*-axis and the *y*-axis counterclockwise around the *z*-axis by a *y* angle. At this time, the *x*-axis coincides with the *X*-axis, the *y*-axis coincides with the axis, and a rotation variable *R*_*z*_(*γ*) is generated, whose matrix is shown in the following formula:

(4) $$\begin{array}{c} R_{z}\left(\gamma \right)=\left[\begin{array}{ccc} \cos\;\gamma & -\sin\;\gamma & 0\\ \sin\;\gamma & \cos\;\gamma & 0\\ 0 & 0 & 1 \end{array}\right]. \end{array}$$

The whole process consists of three independent rotations, generating a rotation matrix *R*, where *R* is the product of *R*_*z*_(*α*) , *R*_*x*_(*α*), and *R*_*z*_(*γ*), and the calculation result is shown in the following formula:

(5) $$\begin{array}{c} R=\left[\begin{array}{ccc} \cos\;\alpha \;\cos\;\gamma -\cos\;\beta \;\sin\;\alpha \;\sin\;\gamma & -\cos\;\beta \;\cos\;\gamma \;\sin\;\alpha -\cos\;\alpha \;\sin\;\gamma & \sin\;\alpha \;\sin\;\beta \\ \cos\;\gamma \;\sin\;\alpha +\cos\;\alpha \;\cos\;\beta \;\sin\;\gamma & \cos\;\alpha \;\cos\;\beta \;\cos\;\gamma -\sin\;\alpha \;\sin\;\gamma & -\cos\;\alpha \;\sin\;\beta \\ \sin\;\beta \;\sin\;\gamma & \cos\;\gamma \;\sin\;\beta & \cos\;\beta \end{array}\right]. \end{array}$$

As shown in formula (6), the two vectors (*x*_1_, *y*_1_, *z*_1_) and (*x*_2_, *y*_2_, *z*_2_) in the above two camera coordinate systems can be converted to each other under the action of the rotation matrix R. It can be seen that the rotation-translation matrix is invertible, and the inverse matrix of the rotation matrix is its own transpose matrix, as shown in the following formula:

(6) $$\begin{array}{c} \left[\begin{array}{c} -x_{2}\\ -y_{2}\\ -z_{2} \end{array}\right]R=\left[\begin{array}{c} x_{1}\\ y_{1}\\ z_{1} \end{array}\right], \end{array}$$

(7) $$\begin{array}{c} R^{-1}=R^{T}. \end{array}$$

When multiple cameras are calibrated, due to the accuracy of target positioning and the minimization of the global error of the pursuit, as shown in formula (8), there is a very small error in the external parameters, and the same point corresponds to different coordinates in the two camera coordinate systems. Among them, the coordinate of this point in a camera coordinate is (*x*_*m*_, *y*_*m*_, *z*_*m*_). The other camera coordinate system is converted to this camera coordinate system through the rotation and translation matrix after calibration. The corresponding coordinate is (*x*_*n*_, *y*_*n*_, *z*_*n*_). Because it is the same point in the real space, it should coincide in the unified coordinate system. If there is no coincidence, there must be an error in the rotation and translation matrix. Therefore, this error needs to be analyzed.

If the origin of the camera coordinate system coincides after the transformation, according to the coordinate transformation process, the relative position of the points in the same coordinate system does not change, then the distance between the two points and the origin should be equal. At this time, the point coordinate can be regarded as a vector; that is, the vector's modulus is equal, as shown in the following formula:

(8) $$\begin{array}{c} x_{m}^{2}+y_{m}^{2}+z_{m}^{2}=x_{n}^{2}+y_{n}^{2}+z_{n}^{2}. \end{array}$$

When the origins are coincident, there is no need to retranslate the camera coordinate system, and only the parameters in the rotation matrix need to be calculated accurately. Finally, the translation vector is transformed once using (6) to reduce the error. Therefore, there is a very small angular rotation between the two coordinates, and the camera coordinate system that needs to be aligned is rotated by three angles in turn according to the rotation method shown in Figure 2.

We assume that the vectors (*x*_*m*_, *y*_*m*_, *z*_*m*_) and (*x*_*n*_, *y*_*n*_, *z*_*n*_) form a small angle *θ*. The relationship between the included angle *θ* and the two vectors is shown in the following formula:

(9) $$\begin{array}{c} \sin\frac{\theta }{2}=\frac{\sqrt{{\left(x_{m}-x_{n}\right)}^{2}+{\left(y_{m}-y_{n}\right)}^{2}+{\left(z_{m}-z_{n}\right)}^{2}}}{2\sqrt{x_{m}^{2}+y_{m}^{2}+z_{m}^{2}}}. \end{array}$$

When the included angle *θ* is very small and approaches 0, the values of *θ* and sin  *θ* are equal, and its geometric meaning is the ratio of the distance between the two points to the vector modulus. Because the viewing angle of the camera is limited to a certain extent, in order to obtain a wider field of view, the camera will be arranged in a relatively far position, and the modulus of the camera coordinate vector will also increase accordingly. Moreover, the common field of view formed by binocular imaging is further compressed, and the measurement area will be divided into multiple segments, which will cause the measurement points in other camera coordinate systems to be converted into the global coordinate system and cause great errors. In order to avoid great errors, further optimization of the calibration external parameters must be carried out.

Another case is when the lengths of the two vectors (*x*_*m*_, *y*_*m*_, *z*_*m*_) and (*x*_*n*_, *y*_*n*_, *z*_*n*_) are not equal. Then, the relationship between the included angle *θ* and the two vectors is shown in the following formula:

(10) $$\begin{array}{c} \cos\;\theta =\frac{\left|x_{m}x_{n}+y_{m}y_{n}+z_{m}z_{n}\right|}{\sqrt{x_{m}^{2}+y_{m}^{2}+z_{m}^{2}}+\sqrt{x_{n}^{2}+y_{n}^{2}+z_{n}^{2}}}. \end{array}$$

At this time, it is not only the rotation angle that causes the camera coordinate system error but also the translation vector error. The allowable range of this error should consider the difference between the lengths of the two vectors and the distance between the two measurement points. In general, the optimization of the overall parameters of the camera during measurement will rarely cause a large change in the translation vector; that is, the distance between the origins of the two coordinate systems will not be very far. Therefore, the vector length difference will not exceed the measurement spread.

**Figure 3:**
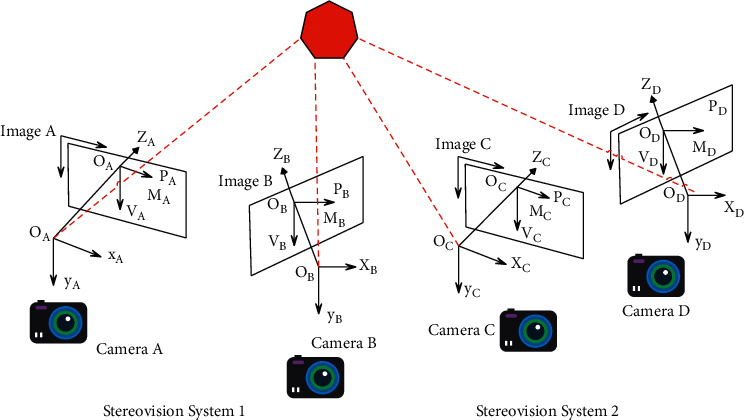
Schematic diagram of coordinate system transformation in a multicamera system.

The conversion relationship between the two coordinate systems is calculated and obtained according to the geometric relationship between the coordinate systems of each camera in the process of constructing a large field of view in a multicamera system. Global calibration is to calibrate the overall measurement system to obtain the conversion relationship between all camera coordinate systems and reference coordinate systems. Taking a dual-camera station system composed of four cameras as an example, the multicamera global calibration process is described in detail with reference to Figure 3.

According to the definition of each coordinate system, it can be known that the transformation relationship between the camera coordinate systems of each camera in the entire measurement system is as follows:

The transformation of the A camera coordinate system and the B camera coordinate system of the measurement system 1 is as follows:

(11) $$\begin{array}{c} \left[\begin{array}{c} x_{A}\\ y_{A}\\ z_{A} \end{array}\right]R_{BA}=\left[\begin{array}{c} x_{B}\\ y_{B}\\ z_{B} \end{array}\right]+T_{BA}. \end{array}$$

The conversion of the *c* camera coordinate system of the measurement system 2 to the *D* camera coordinate system is as follows:

(12) $$\begin{array}{c} \left[\begin{array}{c} x_{C}\\ y_{C}\\ z_{C} \end{array}\right]R_{DC}=\left[\begin{array}{c} x_{D}\\ y_{D}\\ z_{D} \end{array}\right]+T_{DC}. \end{array}$$

In the above, *R*_*BA*_ and *T*_*BA*_, *R*_*DC*_ and *T*_*DC*_ can be directly solved by the camera calibration principle. Among them, the *R* matrix and the *T* matrix can complete the inverse transformation of the two coordinate systems by formulas (6) and (7). In order to realize the coordinate conversion between the two measurement systems, the rotation matrix *R*_*CA*_ and translation vector *T*_*CA*_ of the A camera coordinate system and the C camera coordinate are directly solved by calibration. It can be solved indirectly by C camera calibration and B camera calibration rotation matrix *R*_*CB*_ and translation vector *T*_*CB*_, or by constructing *D* camera and A camera calibration parameters rotation matrix *R*_*DA*_ and translation vector *T*_*DA*_.

(13) $$\begin{array}{c} R_{CA}=R_{BA}R_{CB},\\ R_{CA}=R_{DA}R_{CD},\\ T_{CA}=R_{DA}+T_{CD}+T_{DA},\\ T_{CA}=R_{BA}+T_{CB}+T_{BA}. \end{array}$$

The same target point exists in the common field of view, and the relationship between multiple target points *P*_*A*_(*x*_*Ai*_, *y*_*Ai*_, *z*_*Ai*_) in the A camera coordinate system and the corresponding target point *P*_*C*_(*x*_*Ci*_, *y*_*Ci*_, *z*_*Ci*_) in the C camera coordinate system is constructed, where *i* = 1,2,3, .... The parameters in the transformation matrix and transformation vector that satisfy multiple target points are solved.

(14) $$\begin{array}{c} \left[\begin{array}{c} x_{Ai}\\ y_{Ai}\\ z_{Ai} \end{array}\right]=\left[\begin{array}{ccc} r_{1} & r_{2} & r_{3}\\ r_{4} & r_{5} & r_{6}\\ r_{7} & r_{8} & r_{9} \end{array}\right]\left[\begin{array}{c} x_{Ci}\\ y_{Ci}\\ z_{Ci} \end{array}\right]+\left[\begin{array}{c} t_{x}\\ t_{y}\\ t_{z} \end{array}\right]. \end{array}$$

If it is assumed that there is a matrix A, then *A*^*T*^ represents the transposed matrix of A. Similarly, ‖*A*‖_2_ and ‖*A*‖_*∞*_ represent the spectral norm and row sum norm of matrix A, respectively. The singular values of matrix A are to be solved, the largest of which is the spectral norm of the matrix A. The sum of the absolute values of the elements in each row of matrix A is calculated, the largest of which is the row sum norm of matrix A.

f is a functional relationship that maps the parameter vector *P* ∈ *R*^*m*^ to the estimated measurement vector $\hat{x}=f\left(p\right),\hat{x}\in R^{m}$. An initial estimated parameter *p* and a measurement vector *x* are provided, and it is expected to find *p* + the vector *f* that best satisfies the functional relationship, that is, minimize the squared distance *ε*^*T*^*ε* , where $\varepsilon =x-\hat{x}$. The basis of the LM algorithm is to solve a linear approximation *f* in the neighborhood of *δ*_*p*_. For a very small value of *d*, the Taylor series expansion approximation is as follows:

(15) $$\begin{array}{c} f\left(p+\delta_{p}\right)\approx f\left(p\right)+J\delta_{p}. \end{array}$$

Among them, *J* is the Jacobian matrix ∂*f*(*p*)/∂*p* . Like all nonlinear substitution methods, LM is iterative, starting from a chosen starting point *p*_0_. This method generates a series of vectors *p*_1_, *p*_2_, *p*_3,..._. These vectors converge to the local minima *p*+ with respect to the functional relation *f*. Therefore, at each step, it is necessary to find a suitable value *δ*_*p*_ to minimize the overall value ‖*x* − *f*(*p*+*δ*_*p*_)‖ ≈ ‖*x* − *f*(*p*) − *Jδ*_*p*_‖=‖*ε* − *Jδ*_*p*_‖, and finding *δ*_*p*_ is the solution to a linear least-squares problem. When the column space of *Jδ*_*p*_ − ∈ and *J* is orthogonal, *δ*_*p*_ reaches the minimum value *z* and *fJ*^*T*^(*Jδ*_*p*_ − *ε*)=0 can be obtained, and the resulting value 6 can be used as the solution of the normal equation.

(16) $$\begin{array}{c} J^{T}J\delta_{p}=J^{T}\varepsilon . \end{array}$$

The *J*^*T*^*J* in the matrix on the left side of the equation is an approximation to the Hessian matrix, that is, to the second derivative matrix. The LM method actually solves for small changes in the equation, which can be called augmented regular equations.

(17) $$\begin{array}{c} N\delta_{p}=J^{T}\varepsilon . \end{array}$$

Among them, the off-diagonal elements of N are the same as the corresponding elements in *J*^*T*^*J*, and its diagonal elements are *N*_*ii*_=*μ*+[*J*^*T*^*J*]_*ii*_, where *μ* > 0. The process of changing the diagonal elements of *J*^*T*^*J* is called damping, and *μ* is the damping factor. In the iterative process, if a new parameter vector *p*+*δ*_*p*_ is obtained, which reduces the error *ε*, where *δ*_*p*_ can be calculated by the equation, the new parameter vector can be made closer to the optimal solution. Moreover, the iterative process is repeated with the aim of reducing the damping value.

If the new parameter vector *p*+*δ*_*p*_ is obtained and the error *ε* becomes larger or does not change, the damping value is increased, the augmented canonical equation is solved again, and the algorithm iterates until a value *δ*_*p*_ that reduces the error is found. In the equation, the solution is repeated for different damping factors *μ* until a parameter vector *p*+*δ*_*p*_ that is closer to the approximation is found. This update process corresponds to one iteration in the LM algorithm.

In the LM algorithm, the damping factor *μ* is adjusted during each iteration to ensure that the error can be reduced. If damping is set to a large value *f*, the matrix N in the equation used is almost on the diagonal and the LM update step value *δ*_*p*_ is close to the direction of the steepest descent. Also, the value of *δ*_*p*_ decreases in this case. Damping also handles the case of insufficient Jacobian elements, thus making *J*^*T*^*J* a singular matrix.

The LM algorithm terminates when at least one of the following conditions is met:

(1) The gradient size of *ε*^*T*^*ε* and *Jε* on the right side of the equation will drop below a threshold.
(2) The relative change of the size of the step value *δ*_*p*_ falls below another threshold *ε*_2_.
(3) The error *ε*^*T*^*ε* falls below another threshold *ε*_3_.
(4) The maximum number of iterations *k*_max_ is completed.
  (18) $$\begin{array}{c} J^{T}\sum_{x}^{-1}J\delta_{p}=J^{T}\sum_{x}^{-1}\varepsilon . \end{array}$$
If the covariance matrix Σ_*x*_ for the measurement vector *x* is available, ∑_*x*_^−1^ and the norm *ε*^*T*^∑_*x*_^−1^*ε* can be incorporated into the LM algorithm by minimizing squares instead of directly solving for *ε*^*T*^*ε*. Therefore, a least-squares problem with minimum weights defined by weighted canonical equations is solved.
(4) Research on multicamera photography image art in BERT motion is based on the deep learning model

**Figure 4:**
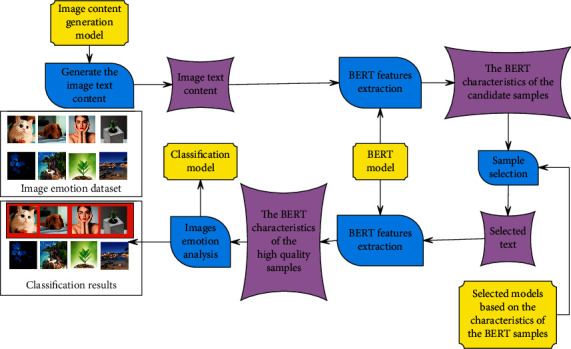
Image sentiment analysis process based on BERT feature sample selection and image content generation.

The image sentiment analysis model of sample selection and image content generation based on BERT features includes four parts: “image content generation,” “text feature extraction,” “sample selection based on BERT features,” and “image sentiment analysis.“ The image is processed through deep learning, as shown in Figure 4.

**Figure 5:**
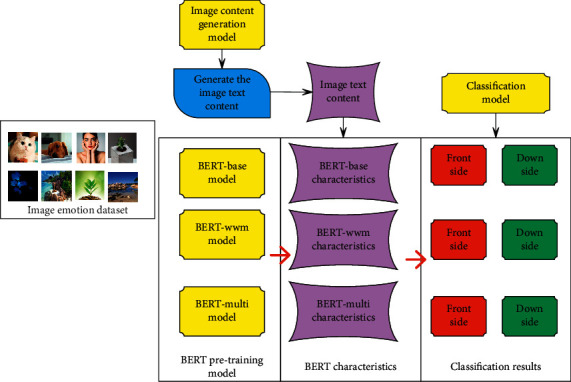
Flowchart of the use of the BERT model.

The specific process of image content generation is shown in Figure 5.

**Figure 6:**
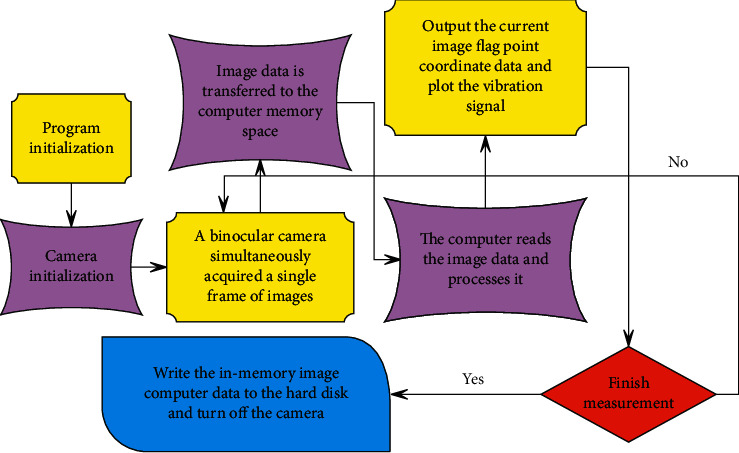
Program flowchart.

Figure 6 shows the flowchart of the binocular stereo vision measurement procedure. The binocular cameras are synchronously triggered to capture a frame of pictures and then transfer them to the computer memory through the USB 3.0 interface. Then, it calls the image data to perform a series of tasks such as marker point detection and image point matching with the same name, then solves the pixel coordinates of the center of the marker point, and then performs 3D reconstruction to obtain the coordinates of the marker point in physical space and output the data.

**Figure 7:**
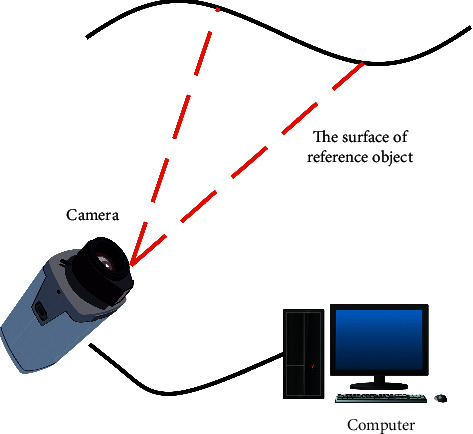
Schematic diagram of camera pose measurement system.

According to the actual measurement needs, a CCD camera bracket and a fixed platform, lighting equipment, etc. are added to the system as auxiliary. The connection diagram of the main part of the system is shown in Figure 7.

**Figure 8:**
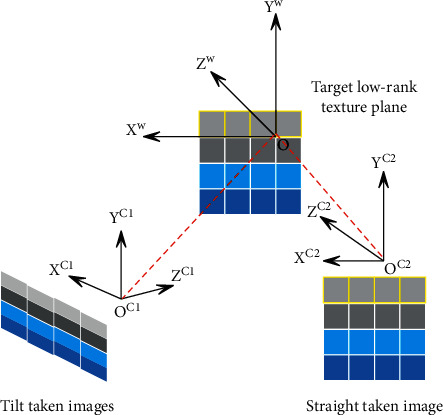
Front-facing images shot and oblique shot images.

The camera coordinate system and the imaging plane coordinate system are established. As shown in Figure 8, the world coordinate system in this section is selected on the target plane containing low-rank textures. For the convenience of description, the following two definitions are made first. When there is no rotation of the camera imaging plane coordinate system relative to the xw0wYw plane of the world coordinate system, the image captured at this time is called a facing image. When the camera imaging plane coordinate system and XwDwYw, the plane has some unknown rotation and translation, the image captured at this time is called an oblique captured image. Obviously, compared with the original low-rank texture image, the front-facing image has no deformation, only scaling, and still retains the low-rank characteristic. When shooting at an angle, the image no longer retains low-rank properties due to projection distortion.

On the basis of the above research, the system model proposed in this article is verified, and the expression effect and image art of the multicamera image are evaluated, and the results shown in Tables 1 and 2 are obtained.

From the above research, it can be seen that the research method of multicamera photography image art in BERT motion based on the deep learning model proposed in this article can effectively improve the expression effect of image art.

## 4. Conclusion

Photography is a kind of visual art, but its expression must be carried out through forms, and different forms bring completely different visual experience. There are many forms of expression, and as one of the visual arts effects, ordering cannot be underestimated. “Ordering” as a guideline in graphic design can make the design more organized and normative. Similarly, for photography, “ordering” will make the photographic work have different formal meanings and show a strong sense of design order. There are many aspects to the formal expression of order in photography, such as symmetry and balance, repetition, and gradual change. These forms of order are often used in graphic art design, which can make the designed picture produce a visual experience of different orders. This article combines the deep learning model to conduct multicamera photographic image art research in BERT motion. The experimental research results show that the research method of multicamera photography image art in BERT motion based on the deep learning mode proposed in this article can effectively improve the expression effect of image art.

## Figures and Tables

**Table 1:** Expression effects of images obtained by multicamera photography.

Num	Image expression
1	88.18
2	88.99
3	89.51
4	92.73
5	93.77
6	93.81
7	90.25
8	93.21
9	95.29
10	94.59
11	89.42
12	90.74
13	94.84
14	91.28
15	93.75
16	89.30
17	90.44
18	90.00
19	88.68
20	95.94
21	93.01
22	95.17
23	95.38
24	89.76
25	92.51
26	94.99
27	94.53
28	90.61
29	95.25
30	91.77
31	90.05
32	89.88
33	90.56
34	89.46
35	89.66
36	90.74
37	91.43
38	92.86
39	95.43
40	89.85
41	94.19
42	95.16
43	94.74
44	88.54
45	91.94
46	95.36
47	88.43
48	93.64
49	95.12
50	89.44
51	95.78

**Table 2:** Artistic effects of images obtained by multicamera photography.

Num	Graphic arts
1	84.07
2	83.00
3	90.76
4	90.02
5	92.86
6	83.46
7	83.77
8	91.17
9	92.85
10	88.45
11	85.95
12	83.05
13	82.56
14	89.53
15	91.53
16	88.21
17	91.44
18	92.89
19	85.75
20	91.94
21	91.70
22	88.77
23	82.79
24	86.10
25	86.03
26	87.87
27	87.52
28	85.69
29	88.45
30	86.89
31	92.98
32	85.11
33	83.83
34	88.94
35	87.49
36	84.71
37	83.35
38	92.65
39	89.39
40	92.69
41	90.92
42	90.18
43	87.85
44	82.91
45	90.89
46	88.62
47	84.07
48	84.70
49	85.80
50	91.05
51	90.76

## Data Availability

The labeled datasets used to support the findings of this study are available from the corresponding author upon request.
